# Prevalence and Predictors of Anxiety and Depressive Symptoms Among International Medical Students in China During COVID-19 Pandemic

**DOI:** 10.3389/fpsyt.2021.761964

**Published:** 2021-11-04

**Authors:** Lu-Lu Yuan, Lu Lu, Xue-Hang Wang, Xiao-Xi Guo, Hong Ren, Yu-Qin Gao, Bo-Chen Pan

**Affiliations:** ^1^Center for Reproductive Medicine, Department of Obstetrics and Gynecology, Shengjing Hospital of China Medical University, Shenyang, China; ^2^Liaoning Provincial Key Laboratory of Oral Diseases, School and Hospital of Stomatology, China Medical University, Shenyang, China; ^3^Department of Teaching Affairs, China Medical University-The Queen's University of Belfast Joint College, China Medical University, Shenyang, China; ^4^International Education School, China Medical University, Shenyang, China

**Keywords:** COVID-19, international medical students, mental health, anxiety symptoms, depressive symptoms, perceived stress

## Abstract

**Background:** The rapid spread of Coronavirus Disease-19 (COVID-19) infection has been the most important public health crisis across the globe since the end of 2019. Anxiety and depression are the most common mental health problems among people during the pandemic, and many studies have reported anxiety and depressive symptoms in college students. However, information on the mental health status of international medical students during this critical period of time has been scarce, which hinders the efforts in making proper policy or strategies to help these students. The present study aims to explore the prevalence of anxiety and depressive symptoms in international medical students in China and to find out the factors that have potential predictive value for anxiety and depressive symptoms.

**Method:** A cross-sectional study was carried out for international medical students during November 2020 at China Medical University in Shenyang, China. Five hundred and nineteen international students were interviewed with questionnaires containing demographic variables, Stressors in school, Generalized Anxiety Disorder Assessment (GAD-7), Patient Health Questionnaire-9 (PHQ-9), Simplified Coping Style Questionnaire (SCSQ), Perceived Stress Scale (PSS-10), the Multidimensional Scale of Perceived Social Support (MSPSS), Revised Life Orientation Test (LOT-R) and Resilience Scale-14 (RS-14). Univariate logistic regression and stepwise multiple logistic regression analyses were conducted where appropriate to explore the predictive factors of anxiety symptoms and depressive symptoms.

**Results:** The prevalence of anxiety symptoms and depressive symptoms in the sample population was 28.5% (148/519) and 31.6% (164/519), respectively. Stressors in school (β = 0.176, OR = 1.192, CI: 1.102–1.289), negative coping style (β = 0.639, OR = 1.894, CI: 1.287–2.788) and perceived stress (β = 0.230, OR = 1.258, CI: 1.184–1.337) were found to be the predictors of anxiety symptoms among the international medical students; while gender (β = −0.594, OR = 0.552, CI: 0.315–0.968), stay up late (β = 0.828, OR = 2.288, CI: 1.182–4.431), current place of residence (β = 1.082, OR = 2.951, CI: 1.256–6.931), stressors in the school (β = 0.303, OR = 1.354, CI: 1.266–1.496), negative coping style (β = 0.866, OR = 2.377, CI: 1.516–3.725), perceived stress (β = 0.233, OR = 1.262, CI: 1.180–1.351) were found to be predictors of depressive symptoms.

**Conclusion:** The prevalence of anxiety symptoms and depressive symptoms was moderate among international medical students in China. The communal predictors of anxiety and depressive symptoms were stressors in school, negative coping style and perceived stress; while demographic factors such as gender (male), stay up late at night and current place of residence were found associated with depressive symptoms. These results suggest that proper stress management and specific interventions are needed to help students maintain their mental health during the COVID-19 pandemic period.

## Introduction

The rapid spread and frequent resurgence of COVID-19 infection have become the most urgent public health issue across the globe since the end of 2019. As of the beginning of August 2021, over 198 million COVID-19 infections were recorded globally claiming the lives of over 4.2 million people (1). World Health Organization (WHO) has announced the COVID-19 outbreak as a pandemic in 2020 (2). In this situation, governments have been encouraged to establish measures to reduce transmission of COVID-19 from person to person, and a wide range of control measures have been implemented (3). In China, the outbreak of COVID-19 happened in early 2020. The Chinese government took strict measures and quickly brought the outbreak under control. In the meantime, the Ministry of Education of China took actions to guide and require colleges and universities to fully launch online teaching and other teaching measures (4). Although the measures adopted during the epidemic have been effective to various extents, they have greatly impacted on all aspects of the population including the mental health across the globe at the same time (5). Many stressors have been identified such as the fear of exposure to infection, worry of family members and loved ones being infected, fear of relative's death, prolonged quarantine, financial strains, limited physical activities, restricted social entertainment, and sense of loneliness, uncertainty and insecurity (5, 6).

Higher education itself for some college students can be a challenge, and many studies have reported a high prevalence of anxiety and depression in students (7, 8). This is especially true for medical students: although a few studies did not find a higher prevalence of mental health problems in medical students (9), more recent studies have reported that medical students experienced more anxiety and depression compared with students of other subjects (10–12). In particular, the conditions of international medical students deserve special attention. One obvious reason is the trans-cultural adaptation issues such as language barrier and cultural differences which sometimes can be stressful to the international students (13). In addition, because of the pandemic of COVID-19, many international students have been stuck in their residential areas or home countries and can only attend online courses which may become difficult due to time differences or internet accessibility problems (14). Furthermore, clinical rotation and clerkship are a crucial part of medical education (15, 16), but in some cases, this becomes unobtainable for some international students. We could anticipate that stress from the above-mentioned sources may result in mental health problems. However, little study has been done in this area.

Several factors are known related to the occurrence of anxiety and depression among students. For example, age, gender, and education levels had significant associations with anxiety/depressive symptoms among students (7, 8, 13, 14). Coping which was defined as the cognitive attempts and behavioral adaption to deal with stressors (17) was found related to the mental health problems, and people with negative coping style tended to have adverse outcomes (18). Perceived stress is people's self-assessment of the threat from stressors, and has been shown associated with anxiety and depressive symptoms in many studies (19–21). Furthermore, psychological resources such as social support, optimism and resilience may also play some roles. Social support is defined as the material or moral support provided to the individuals under stress or in a difficult condition by the people around him/her. It contributes to the development of individuals' behavioral patterns, social cognition and values (22). Optimism is a personality trait characterized by a general tendency to hold positive expectations about the future; it functions as a psychological resource conferring health benefits (23). Finally, resilience is a person's ability to grow from dealing with stressors or adverse changes (24). All these psychological resources have been shown to have positive effects on anxiety and depression in students (19, 25–28). When dealing with stress and its related mental health problems, it is important to explore these relevant psychosocial factors so that we can provide essential psychological support to the students to alleviate their anxiety and depressive symptoms. However, so far little investigation has been done to explore their roles in the mental health of international medical students. We hypothesize that negative coping style and perceived stress are positively associated with anxiety and depressive symptoms while positive coping style, perceived social support, optimism, and resilience are negatively associated with the symptoms. The aim of the current study is to first explore the prevalence of anxiety and depressive symptoms in international medical students in China, and then find out the factors that have potential predictive value for these symptoms.

## Materials and Methods

### Settings of the Study

This cross-sectional study on international students was conducted at China Medical University. Data were collected online during November 2020. The research was approved by the Research Ethics Committee at China Medical University.

### Subjects

All participates were international medical students from China Medical University. The inclusion criteria were: (1) a current student of the University, (2) can get access to the internet, (3) can read and understand the survey content.

### Procedure

**Figure 1:**
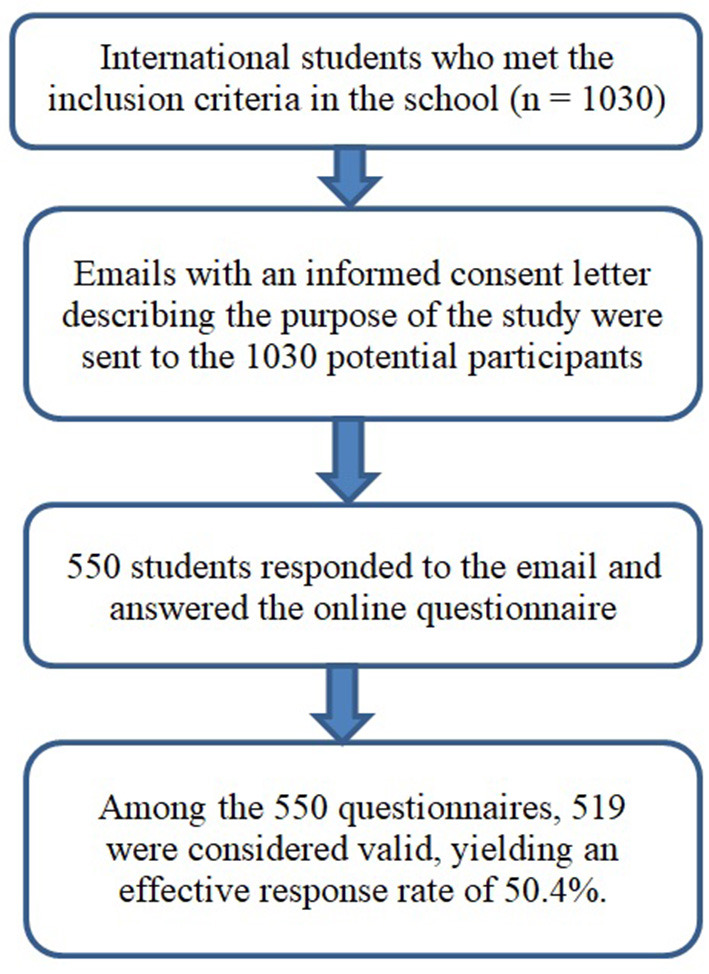
Flow chart of the data collection process.

The whole process of the study was anonymous and voluntary for respondents. Emails describing the purpose of the study were sent to the potential participants by their class advisors. The emails contained the link for the students to access the online questionnaire, and they were sent out to all the eligible students including undergraduate and postgraduate students. When the participants visited the website to answer the online questionnaire, they were greeted with an informed consent letter stating that the survey was completely voluntary. In the end, 550 students completed the questionnaire. The detailed process of sending and collecting questionnaires is described in Figure 1.

### Tools

Demographic characteristics were recorded with a general questionnaire which included age, gender, educational background, current place of residence, COVID-19 outbreak in the city, residence style, smoking, drinking alcohol, exercise, stay up late, and addicted to the Internet. Stressors in school among international students were assessed in the following seven aspects: academic difficulties, language barrier, interpersonal relation difficulties, health problems, financial pressure, daily life difficulties, and adverse life events.

### Measurement of Anxiety Symptoms

Generalized Anxiety Disorder Assessment (GAD-7) (29) was used to assess the anxiety symptoms of the international students. The GAD-7 included 7 items, and each item was rated on a 4-point scale (0–3), with a total score ranging from 0 to 21. A higher score means more severe anxiety symptoms. GAD-7 has been reported with good reliability and validity (30). The Cronbach's α was 0.92 in the current study.

### Measurement of Depressive Symptoms

Depressive symptoms were assessed with the Patient Health Questionnaire-9 (PHQ-9) (31). The PHQ-9 is a 9-item tool rating on 4-point scoring system (0–3) with a total score ranging from 0 to 27. A total score of 5 or above was considered depressive tendency. The version has been shown with good reliability and validity (32). The Cronbach's α was 0.90 in the current study.

### Measurement of Coping Style

Coping Style was assessed by the Simplified Coping Style Questionnaire (SCSQ) (33). The SCSQ is a 20-item scale with 2 domains: positive coping style and negative coping style. The positive coping style reflected positive coping strategies, such as “looking for suggestions from relatives, friends or classmates.” The negative coping style reflected negative coping strategies, such as “relying on somebody else to solve the problem.” Each item was scored on a 4-point scale (0–3). A higher domain score reflected a preference for adopting the relevant coping style. The scale had been found with good reliability and validity (34, 35). In the current study, the Cronbach's α was found to be 0.91.

### Measurement of Perceived Stress

The level of perceived stress was assessed by the 10-item version of Perceived Stress Scale (PSS-10) (36). Each item was scored using a 5-point scale, with a total score ranging from 0 to 40. Higher scores indicated a higher level of perceived stress. The PSS-10 has demonstrated good reliability and validity (37). The Cronbach's α was 0.87 in this study.

### Measurement of Social Support

The Multidimensional Scale of Perceived Social Support (MSPSS) was used to assess the level of perceived social support for the international medical students (38). It measured perceived support from three social relationships: family, friends and significant others (such as relatives or schoolmates). The MSPSS was a 12-item scale rated on a 7-point scale. The total score ranged from 12 to 84, with a higher score indicating higher social support. The scale had good reliability and validity (39, 40). In this study, the Cronbach's α of the MSPSS was 0.94.

### Measurement of Optimism

Optimism was assessed by the 10-item Revised Life Orientation Test (LOT-R) (23). It consisted of ten items using a 5-point rating system, three of which were for optimism; three were for pessimism; the other four items served as fillers. The Cronbach's α was 0.71 in the current research.

### Measurement of Resilience

Respondents' resilience was measured with the Resilience Scale-14 (RS-14) (41). The RS-14 included 14 items, and each item was rated on a 7-point scale, with a total score ranging from 14 to 98. Higher scores indicated higher levels of resilience. RS-14 had been used in previous studies, and the reliability and validity had been confirmed (42, 43). The Cronbach's alpha coefficient for the total scale of resilience was 0.96 in the present study.

### Operational Definition

The cut-off points of GAD-7 and PHQ-9 were set to differentiate whether the international students had anxiety or depression symptoms, respectively. According to the previous studies (30, 31), students with a GAD-7 score at or above 5 were divided into the anxiety symptoms group, and students with a PHQ-9 score at or above 5 were categorized into the depressive symptoms group.

### Statistical Analyses

Statistical Package for Social Sciences (SPSS 20.0 for Windows) was used to conduct data analyses. Significance for all statistical tests was set at the level of 0.05 (two-tailed). Univariate logistic regression was used to explore the relationship between anxiety/depressive symptoms and the categorical demographic variables /continuous variables. Stepwise multiple logistic regression analyses were conducted to find the predictors. The variables with *p* < 0.2 in the univariate logistic regression were entered into regression analysis in order not to overfit the logistic regression models (44). Variables were entered in the regression analysis at *p* < 0.05 and removed from the model at *p* > 0.10. Variables with *p* < 0.05 were not included in the tables of logistic regression. Data provided in the regression models included regression coefficient (β), OR, and 95% CI.

## Results

### Descriptive Statistics

In the current study, 550 questionnaires were collected. Among them, 519 were considered valid, yielding an effective response rate of 50.4%. Altogether 276 male and 243 female students participated.

All in all, 148 students reported anxiety symptoms; 164 reported depressive symptoms; and the prevalence was 28.5 and 31.6%, respectively. The demographic information of the participants was described in Table 1. The median (IQR) age of the respondents was 22 (3) years, ranging from 16 to 42. Most participants (*n* = 453, 87.3%) were undergraduates; 86% (*n* = 451) of the students were not in China at the time of the questionnaire survey. Most respondents (*n* = 409, 78.8%) were experiencing the COVID-19 pandemic.

**Table 1:** Distributions of anxiety symptoms and depressive symptoms in categorical demographic variables (*n* = 519).

	***N*** **(%)**	**Anxiety**	**Depressive**
		**symptoms**	**symptoms**
		***N*** **(%)**	***N*** **(%)**
**Gender**			
Male	276 (53.2)	79 (28.6)	94 (34.1)
Female	243 (46.8)	69 (28.4)	70 (28.8)
**Educational background**			
Undergraduate	453 (87.3)	134 (29.6)	150 (33.1)
Master	32 (6.2)	5 (15.6)	4 (12.5)
Doctorate	27 (5.2)	8 (29.6)	8 (29.6)
Trainees	7 (1.3)	1 (14.3)	2 (28.6)
**Current place of residence**			
China	68 (13.1)	22 (32.4)	26 (38.2)
Asia outside China	376 (72.4)	111 (29.5)	124 (33.0)
Other continents	75 (14.5)	15 (20.0)	14 (18.7)
**Outbreak in the city**			
No	110 (21.2)	19 (17.3)	23 (20.9)
Yes	409 (78.8)	129 (31.5)	141 (34.5)
**Residence style**			
Live alone	170 (32.8)	53 (31.2)	68 (40.0)
Live with family or friends	349 (67.2)	95 (27.2)	96 (27.5)
**Smoking**			
No	496 (95.6)	140 (28.2)	153 (30.8)
Yes	23 (4.4)	8 (34.8)	11 (747.8)
**Drinking alcohol**			
No	498 (96.0)	141 (28.3)	155 (31.1)
Yes	21 (4.0)	7 (33.3)	9 (42.9)
**Exercise**			
No	59 (11.4)	20 (33.9)	22 (37.3)
Yes	460 (88.6)	128 (27.8)	142 (30.9)
**Stay up late**			
No	149 (28.7)	25 (16.8)	24 (16.1)
Yes	370 (71.3)	123 (33.2)	140 (37.8)
**Addicted to the Internet**			
No	105 (20.2)	17 (16.2)	14 (13.3)
Yes	414 (79.8)	131 (31.6)	150 (36.2)

*N, number*.

### Distributions of Anxiety and Depressive Symptoms in Categorical and Continuous Variables

The distributions of anxiety symptoms and depressive symptoms in categorical and continuous variables (age, stressors in school, positive coping style, negative coping style, perceived stress, perceived social support, optimism, and resilience) were presented in Tables 2, 3. Results showed that the distribution of anxiety symptoms were significantly different in some categorical variables (current place of residence, outbreak in the city, stay up late, addicted to the Internet) and all the continuous variables; depressive symptoms were significantly different in some categorical variables (gender, educational background, current place of residence, outbreak in the city, stay up late, addicted to the Internet, smoking, residence style) and all the continuous variables except for positive coping style.

**Table 2:** Univariate logistic regression analysis on results of anxiety symptoms (*n* = 519).

	**β**	**S.E**	**Wals**	***P***	**OR (95%CI)**
**Gender**	−0.011	0.195	0.003	0.954	0.989 (0.675,1.449)
**Educational background**			3.392	0.335	
Undergraduate vs. Trainees	0.924	1.085	0.726	0.394	2.520 (0.301,21.137)
Master vs. Trainees	0.105	1.185	0.008	0.929	1.111 (0.109,11.330)
Doctorate vs. Trainees	0.927	1.159	0.639	0.424	2.526 (0.260,24.513)
**Current place of residence**			3.289	0.193	
China vs. Other continents	0.649	0.388	2.796	0.095	1.913 (0.894,4.092)
Asia outside China vs. Other continents	0.516	0.310	2.771	0.096	1.675 (0.913,3.076)
**Outbreak in the city**	0.791	0.274	8.358	0.004	2.207 (1.290,3.773)
**Residence style**	−0.192	0.205	0.876	0.349	0.826 (0.553,1.233)
**Smoking**	0.305	0.449	0.460	0.497	1.356 (0.562,3.270)
**Drinking alcohol**	0.236	0.473	0.248	0.618	1.266 (0.500,3.202)
**Exercise**	−0.285	0.294	0.941	0.332	0.752 (0.422,1.338)
**Stay up late**	0.904	0.245	13.571	0.000	2.470 (1.527,3.996)
**Addicted to the Internet**	0.874	0.285	9.387	0.002	2.396 (1.370,4.191)
**Age**	−0.043	0.029	2.133	0.144	0.958 (0.905,1.015)
**Stressors in the school**	0.273	0.034	65.055	0.000	1.313 (1.229,1.403)
Health problems	1.319	0.318	17.209	0.000	3.741 (2.006,6.977)
Language barrier	0.530	0.113	21.945	0.000	1.698 (1.361,2.120)
Financial pressure	0.524	0.106	24.701	0.000	1.690 (1.374,2.078)
Academic difficulties	0.713	0.131	29.817	0.000	2.041 (1.580,2.636)
Interpersonal difficulties	1.243	0.169	54.409	0.000	3.467 (2.492,4.825)
Daily life difficulties	0.704	0.125	31.646	0.000	2.023 (1.582,2.585)
Adverse life events	0.589	0.113	27.378	0.000	1.802 (1.445,2.247)
**Positive coping style**	0.242	0.138	3.087	0.079	1.274 (0.972,1.670)
**Negative coping style**	0.887	0.157	31.993	0.000	2.429 (1.786,3.303)
**Perceived stress**	0.288	0.030	89.833	0.000	1.334 (1.257,1.416)
**MSPSS**	−0.015	0.005	7.351	0.007	0.986 (0.975,0.996)
**Optimism**	−0.111	0.027	17.458	0.000	0.895 (0.850,0.943)
**Resilience**	−0.019	0.006	11.727	0.001	0.981 (0.971,0.992)

**Table 3:** Univariate logistic regression analysis on results of depressive symptoms (*n* = 519).

	**β**	**S.E**	**Wals**	***P***	**OR (95%CI)**
**Gender**	−0.244	0.190	1.646	0.200	0.783 (0.540,1.137)
**Educational background**			5,339	0.149	
Undergraduate vs. Trainees	0.213	0.843	0.064	0.800	1.238 (0.237,6.454)
Master vs. Trainees	−1.030	0.993	1.075	0.300	0.357 (0.051,2.500)
Doctorate vs. Trainees	0.051	0.937	0.003	0.956	1.053 (0.168,6.602)
**Current place of residence**			7.244	0.027	
China vs. Other continents	−0.230	0.273	0.709	0.400	0.795 (0.466,1.356)
Asia outside China vs. Other continents	−0.992	0.387	6.560	0.010	0.371 (0.174,0.792)
**Outbreak in the city**	0.688	0.257	7.198	0.007	1.990 (1.204,3.290)
**Residence style**	−0.564	0.197	8.169	0.004	0.569 (0.387,0.838)
**Smoking**	0.720	0.429	2.824	0.093	2.055 (0.887,4.760)
**Drinking alcohol**	0.507	0.451	1.259	0.262	1.660 (0.685,4.021)
**Exercise**	−0.286	0.288	0.992	0.319	0.751 (0.427,1.319)
**Stay up late**	1.154	0.247	21.769	0.000	3.170 (1.953,5.148)
**Addicted to the Internet**	1.306	0.305	18.379	0.000	3.693 (2.032,6.711)
**Age**	−0.61	0.029	4.365	0.037	0.941 (0.888,0.996)
**Stressors in the school**	0.375	0.039	90.284	0.000	1.455 (1.347,1.573)
Health problems	1.365	0.324	17.729	0.000	3.916 (2.074,7.392)
Language barrier	0.646	0.113	32.630	0.000	1.909 (1.529,2.382)
Financial pressure	0.584	0.104	31.371	0.000	1.793 (1.462,2.199)
Academic difficulties	0.960	0.138	48.297	0.000	2.611 (1.992,3.423)
Interpersonal difficulties	1.673	0.194	74.062	0.000	5.326 (3.639,7.796)
Daily life difficulties	0.896	0.130	47.619	0.000	2.449 (1.899,3.159)
Adverse life events	0.794	0.115	47.250	0.000	2.212 (1.764,2.773)
**Positive coping style**	0.098	0.131	0.559	0.455	1.103 (0.853,1.427)
**Negative coping style**	0.856	0.151	32.091	0.000	2.355 (1.751,3.167)
**Perceived stress**	0.281	0.029	91.272	0.000	1.325 (1.251,1.404)
**MSPSS**	−0.016	0.005	9.574	0.002	0.984 (0.974,0.994)
**Optimism**	−0.112	0.026	18.628	0.000	0.894 (0.850,0.941)
**Resilience**	−0.020	0.005	13.184	0.000	0.980 (0.970,0.991)

### Predictors of Anxiety Symptoms and Depressive Symptoms

Stepwise multiple logistic regression analysis was conducted to identify the predictors of anxiety symptoms and depressive symptoms.

Variables that were significantly associated with anxiety symptoms were included in the logistic regression analysis, including demographic variables (age, current place of residence, outbreak in the city, stay up late, addicted to the Internet), stressors in school, positive coping style, negative coping style, perceived stress, perceived social support, optimism, and resilience. Multicollinearity diagnostic tests showed that there was no multicollinearity between the predictor variables. Stepwise multiple logistic regression was conducted and results were shown in Table 4. As a result, stressors in school, negative coping style, and perceived stress were found to be predictors of anxietysymptoms.

**Table 4:** Stepwise multiple logistic regression analysis on results of anxiety symptoms (*n* = 519).

	**β**	**S.E**	**Wals**	***P***	**OR (95%CI)**
Stressors in the school	0.176	0.040	19.339	0.000	1.192 (1.102,1.289)
Negative coping style	0.639	0.197	10.486	0.001	1.894 (1.287,2.788)
Perceived stress	0.230	0.031	55.084	0.000	1.258 (1.184,1.337)
Constant	−8.975	0.866	107.368	0.000	

*Percentile 95% CIs for ORs are defined using the values that mark the upper and lower 2.5% of ORvalue*.

*SE, standard error; CI, confidenceinterval*.

Variables that were significantly associated with depressive symptoms were included in the logistic regression analysis, including demographic variables (age, gender, educational background, current place of residence, outbreak in the city, stay up late, addicted to the Internet, smoking, residence style), stressors in school, negative coping style, perceived stress, perceived social support, optimism, and resilience. Multicollinearity diagnostic tests showed that there was no multicollinearity between the predictor variables. Stepwise multiple logistic regression was conducted and results were shown in Table 5. As a result, gender, stay up late, current place of residence, stressors in the school, negative coping style, perceived stress were found to be predictors of depressive symptoms.

**Table 5:** Stepwise multiple logistic regression analysis on results of depressive symptoms (*n* = 519).

	**β**	**S.E**,	**Wals**	***P***	**OR (95%CI)**
Gender (male vs. female)	−0.594	0.286	4.303	0.038	0.552 (0.315,0.968)
Stay up late	0.828	0.337	6.032	0.014	2.288 (1.182,4.431)
Current place of residence			6.331	0.042	
China vs. Other continents	1.068	0.566	3.560	0.059	2.910 (0.959,8.829)
Asia outside China vs. other continents	1.082	0.436	6.167	0.013	2.951 (1.256,6.931)
Stressors in school	0.303	0.051	35.846	0.000	1.354 (1.266,1.496)
Negative coping style	0.866	0.229	14.252	0.000	2.377 (1.516,3.725)
Perceived stress	0.233	0.034	45.632	0.000	1.262 (1.180,1.351)
Constant	−11.223	2.272	24.404	0.000	

*Percentile 95% CIs for ORs are defined using the values that mark the upper and lower 2.5% of ORvalue*.

*SE, standard error; CI, confidenceinterval*.

## Discussion

### The Prevalence of Anxiety Symptoms and Depressive Symptoms in International Medical Students

The prevalence of anxiety symptoms in the current study was 28.5%. It was lower than that of students of higher education in other subjects in the world (32%) (7) and that of healthcare workers (30%) (45) during the COVID-19 pandemic. This result was perhaps relatively easy to understand, because, in comparison with students of other subjects, medical students may be better prepared in terms of knowledge and skill on combating the pandemic and, to some extent, they may be in a better position to deal with mental health problems than the former. Moreover, unlike healthcare workers, most of the medical students do not have to face the patients directly so that the anxiety experienced by the healthcare workers at the time of pandemic may not be as pronounced in the medical students. On the other hand, with respect to the prevalence of depressive symptoms (31.6%), which was also lower than that of students of higher education in other subjects in the world (34%) (7), it was higher than that of medical students (28%) in the world before the pandemic (9) and that of domestic Chinese university students amid the COVID-19 pandemic (26%) (8), similar to that of healthcare workers during the COVID-19 pandemic (31.1%) (45). This information indicates that the COVID-19 pandemic did have an adverse impact on international medical students' mental health. It also indicates that at the time of a public health crisis such as the COVID-19 pandemic, international students may experience more difficulties than their domestic counterparts due to the trans-cultural differences and poor adaptation. In this sense, it is paramount to find proper intervention strategies to help these students.

### Predictors of Anxiety/Depressive Symptoms in International Medical Students

In the current study, we were able to identify stressors in school, perceived stress and negative coping style as the communal predictors for anxiety symptoms and depressive symptoms, while gender, stay up late and current place of residence were found to be predictors specific for depressive symptoms among international medical students.

According to the results of stepwise multiple logistic regression analysis, “stressors in school” were associated with both anxiety and depressive symptoms, which was consistent with previous studies both during (46) and before (19, 20, 47) the pandemic. In our study, seven stressors were mentioned including academic difficulties, language barrier, interpersonal relation difficulties, health problems, financial pressure, daily life difficulties, and adverse life events. We found that the distribution of anxiety symptoms and depressive symptoms were significantly different in all those aspects (*p* < 0.001). Studies (48) have found that long-term exposure to stressors would increase levels of anxiety and depression among medical students. Therefore, our study results suggest that during the pandemic, while we focus our attention on dealing with mental health issues directly related to the pandemic, the conventional stressors related to school life should not be overlooked. Sometimes, we may have to investigate whether these conventional stressors are aggravated by the pandemic so as to find proper intervention.

Our result that perceived stress made an important contribution to the anxiety and depressive symptoms was also consistent with previous studies both during (49) and before (19, 20) the outbreak in students and other populations (50, 51). In the current study, the only psychological variable in the regression was perceived stress. Perceived stress is people's self-assessment of the threat from stressors as well as their ability to cope with the threat. A moderate level of stress is beneficial and enables the student to become a more dynamic and better performer. However, persistently high levels of stress may cause considerable psychological and physical glitches (20). Thus, the results of our study suggest that it is imperative to identify the students with a high level of stress and the causes, and give them help if necessary. Stress alleviation strategies may involve stressors as aforementioned as well as the ability and assessment. As to the ability to cope with the threat, we can set up some auxiliary courses/lectures in these areas so that students can master some coping skills and improve their ability to cope with the pressure. Similarly, as assessment is an important determinant of adjustment and adaptation to stress (17), students should be given relevant education and counseling. In short, we should educate students to correctly evaluate difficulties and personal abilities, and if the student still can't solve the difficulties, he or she should be encouraged to ask for help from proper sources in time.

Result with the coping style was also consistent with those of previous studies (25, 52). In fact, in our study, the negative coping style was the strongest predictor for anxiety symptoms and the second strongest for the depressive symptoms. Coping was defined as the cognitive attempts and behavioral adaption to deal with stressors. The negative coping style reflects actual negative coping strategies, such as “relying on somebody else to solve the problem.” People with negative coping style tended to have adverse outcomes such as alcohol abuse and suicidal thoughts (18, 53). The strong link of negative coping style with poor mental health of international students in our study should arouse our attention, and students should be given education on how to appropriately cope with the stress or crisis.

On the other hand, it is interesting to note that some of the demographic variables such as gender, stay up late and current place of residence have been demonstrated specifically associated with the depressive symptoms of international students. First, we found that male students had a much higher risk of suffering from depressive symptoms than the female students, which was in discrepancy with results of some previous studies. For example, one study showed that difference in depression between males and females was not statistically significant among medical students (9), and another study found a similar result in students of higher education during the COVID-19 pandemic (7). However, there have been some studies reaching similar conclusions as ours. In particular, one study on overseas medical students during the COVID-19 epidemic in China (14) and one cross-sectional study on domestic medical students in China during the epidemic both showed that male students were more depressed (54), supporting our findings. We speculate that the discrepancy in the reports may be partially due to the special features of the study population. Our study took place during the period when the outbreak of COVID-19 happened in many countries around the world, and the pandemic has caused financial and other crises to many families. Traditionally, Asian male students are expected to be more independent and shoulder more life responsibilities than their female peers. Therefore, they may be more sensitive to the deterioration of family finance or other conditions and under more pressure to deal with situation by them. Indeed, in our study, male students reported more financial pressure (*p* < 0.007) and daily life difficulties (*p* < 0.013) along with academic difficulties (*p* < 0.030) and adverse life events (*p* < 0.005) than the female students did. When the high social expectation and deteriorating situation was coupled with the isolation and inability to deal with the difficulty which was quite a common scenario during the pandemic, more stress and the related depressive symptoms could be anticipated in the male students. Importantly, these results suggest to us that we should pay more attention to the male international students in the school management during this special period of time.

The association of another demographic variable, stay up late, with depressive symptoms came as no surprise to us, because quite many studies have shown that bad lifestyle such as stay up late tend to cause mental health problems in students (55–57). During the COVID-19 outbreak, for most of the time, online courses were the only teaching method. Unlike the offline courses at the university campus when the attendance is usually required, online learning students rely more on self-discipline in their schedule; but when there was local time difference with the school day, students were more likely to stay up late, which made them prone to depression (58). In this sense, in student management, more counseling and instructions are needed to help students develop good study habits and healthy lifestyle.

Finally, current place of residence was found to be an important factor for depressive symptoms, i.e., students staying in Asia outside China had more depressive symptoms compared with other continents. This phenomenon probably reflected the impact of the pandemic: students who stayed in Asia outside China were mainly Indian students, and when this survey was carried out, India was in the outbreak period, which might adversely affect students' mental health status. However, we found that the level of depression among students staying in China was also high. This was somewhat surprising to us, because, when the survey was done, the outbreak of COVID-19 in China already had been brought under control. Accordingly, we had thought that students staying in China might feel safer and therefore have fewer mental health problems than their counterparts staying in other countries. We guess that this finding may be due to several reasons. First, by the time of this study, students who were living in China already had stayed in China for nearly one and half years, so they may become homesick which is commonly seen among international students (59, 60). In addition, they may worry about the safety of their loved ones in their home countries, which is another feature of university students during the pandemic (61). Furthermore, the pandemic prevention measures such as lockdown of the university and the required social distancing at the university campus may lead to a sense of social isolation in the students which may finally result in their depression (5). Nevertheless, this result was very important, because it has reminded us that we should not underestimate the mental health status of the international students who are staying in China, and proper counseling and education on the mental health of these students should be in place.

However, some results of the current study were not consistent with our hypothesis in that positive psychological variables, such as perceived social support, optimism and resilience showed neither significant association with the anxiety symptoms nor with the depressive symptoms. Further research is needed to explore the exact mechanism of those variables and the anxiety/depressive symptoms.

### Strengths and Limitations

The current study aims at identifying the possible predictive factors associated with anxiety and depressive symptoms in international medical students in China. The hypothetical socio-demographic and psychological variables have been analyzed, resulting in significant results. The study is important because it has provided useful information on the mental health status of international medical students during the pandemic, which largely has not been explored before. Some of the results such as the roles of gender, stay up late and current place of residence in the development of depressive symptoms may be pandemic specific or related, which may be instrumental to university authority or educators to properly place their attention and efforts to help the international students. In addition, study results have reminded us that the conventional variables related to mental health such as stressors in school, negative coping style and perceived stress are still very important predictors of student mental health during the pandemic. Therefore, intervention measures for dealing with student mental health problems in these areas would most likely be effective and efficient.

Due to the cross-sectional design, the causal relationship couldn't be confirmed. Future research by means of longitudinal studies should be done to address the relationship. Besides, we only focused on the associations of anxiety/depressive symptoms with stressors, coping style, perceived stress, perceived social support, optimism, and resilience; other factors which may be important to consider for anxiety/depressive symptoms were not included. In addition, “internet addiction” in this study was assessed only by a question instead of a validated instrument, which may affect the validity of the findings. Moreover, a larger and multi-center sample is needed to improve the representativeness of the data. However, despite of these limitations, our study has provided new and useful information on the mental health status of international medical students and suggested potentially effective ways to reduce anxiety and depressive symptoms in the students.

## Conclusion

The prevalence of anxiety and depressive symptoms of the international medical students was moderate. After adjusting for demographic factors, stressors in school, negative coping style and perceived stress were found positively and significantly associated with both anxiety and depressive symptoms. In addition, male gender, “stay up late” and current place of residence were found to be predictors of depressive symptoms. However, positive psychological variables such as perceived social support, optimism and resilience showed neither significant relation with anxiety symptoms nor with depressive symptoms. Study results suggest that at this time of COVID-19 pandemic, situation-specific intervention measures may be potentially effective to help improve the mental health status of the international students.

## Data Availability Statement

The raw data supporting the conclusions of this article will be made available by the authors, without undue reservation.

## Ethics Statement

The studies involving human participants were reviewed and approved by Committee on Human Experimentation of China Medical University. The patients/participants provided their written informed consent to participate in this study.

## Author Contributions

L-LY analyzed the data and drafted the manuscript. LL helped analyzing the statistics. X-HW, X-XG, and HR collected the data. Y-QG helped with the study design and data analysis. B-CP provided the guidance of the study design and reviewed the manuscript. All authors read and approved the final manuscript.

## Conflict of Interest

The authors declare that the research was conducted in the absence of any commercial or financial relationships that could be construed as a potential conflict of interest.

## Publisher's Note

All claims expressed in this article are solely those of the authors and do not necessarily represent those of their affiliated organizations, or those of the publisher, the editors and the reviewers. Any product that may be evaluated in this article, or claim that may be made by its manufacturer, is not guaranteed or endorsed by the publisher.
